# In-silico pre-clinical trials are made possible by a new simple and comprehensive lumbar belt mechanical model based on the Law of Laplace including support deformation and adhesion effects

**DOI:** 10.1371/journal.pone.0212681

**Published:** 2019-03-06

**Authors:** Jérôme Molimard, Rébecca Bonnaire, Woo Suck Han, Reynald Convert, Paul Calmels

**Affiliations:** 1 Mines Saint-Etienne, Univ Lyon, Univ Jean Monnet, INSERM, U 1059 Sainbiose, Centre CIS, Saint-Etienne, France; 2 Thuasne, Saint-Etienne, France; 3 Univ Lyon, UJM Saint-Etienne, LIBM, CHU Saint-Etienne, Physical medicine and rehabilitation, Saint-Etienne, France; Toronto Rehabilitation Institute - UHN, CANADA

## Abstract

Lower back pain is a major public health problem. Despite claims that lumbar belts change spinal posture due to applied pressure on the trunk, no mechanical model has yet been published to prove this treatment. This paper describes a first model for belt design, based on the one hand on the mechanical properties of the fabrics and the belt geometry, and on the other hand on the trunk geometrical and mechanical description. The model provides the estimation of the pressure applied to the trunk, and a unique indicator of the belt mechanical efficiency is proposed: pressure is integrated into a bending moment characterizing the belt delordosing action on the spine. A first in-silico clinical study of belt efficiency for 15 patients with 2 different belts was conducted. Results are very dependent on the body shape: in the case of high BMI patients, the belt effect is significantly decreased, and can be even inverted, increasing the lordosis. The belt stiffness proportionally increases the pressure applied to the trunk, but the influence of the design itself on the bending moment is clearly outlined. Moreover, the belt/trunk interaction, modeled as sticking contact and the specific way patients lock their belts, dramatically modifies the belt action. Finally, even if further developments and tests are still necessary, the model presented in this paper seems suitable for in-silico pre-clinical trials on real body shapes at a design stage.

## Introduction

Among a large series of care strategies for lower back pain, several clinical trials have shown the clinical efficiency of lumbar belts [1, 2]. However, both their mechanical and physiological effects remain unclear. Despite the great number of patients affected every year (A World Health Organization report estimates the lifetime prevalence of non-specific low back pain at 60 to 70% in industrialized countries [3]), few authors have investigated the mechanisms of action of lumbar belts. Most related studies are concerned with braces for scoliosis correction. Beauséjour et al. proposed a link between brace wearing (strap tension), interface pressure and spine correction using the posture changes as a landmark [4]. Recently, authors measured locally the intervertebral disk geometry with a low-dose radiation X-ray [5] or fluoroscopy [6] while wearing a belt, which demonstrated and quantified the induced spine posture changes.

The textile community provided various studies on fabrics for compression or contention medical treatments, such as lumbar belts. Mechanical tests have been used in order to characterise the mechanical properties of fabrics: relaxation or tensile tests [7, 8, 9, 10, 11], sometimes using different speeds [12], or non-uniaxial tensile test [13]. Studies investigated the fabric weaving [14, 15, 16] as well as different manufacturing methods [17] or the effect of physicochemical treatments [18, 19]. They are also used to validate finite element models of fabric [20]. Unfortunately, the link between the textile characteristics, the belt design, the pressure applied on the trunk and its effect on human body and particularly on the spine has been poorly studied.

It was proposed that the main mechanical effect of a lumbar belt is the increased pressure applied on the trunk. This pressure is speculated to act on the abdomen and force the patient to change his or her lumbar spinal posture (lordosis). Many authors investigated the pressure applied by a medical device using pressure sensors. Even if the direct measurement of the pressure on the body is an efficient and relevant way of studying the load transfer between the belt and the trunk, this method implies the complete manufacturing of a new belt and experimentation on manikins or patients [21]. So far, this approach has proved too expensive and time consuming. Recently, Bonnaire [21] proposed a clinical study using optical methods and pressure map sensors, but resorting to a clinical study is a tedious task, and some early stage information would be of great help for the design of new belts. One way to overcome this problem is by developing a finite elements model. A first model based on idealised parametric trunk shape concluded that pressure on lumbar disc is mainly controlled by subject geometrical parameters (size, morphology, lumbar lordosis angle) and belt design (geometry and mechanical properties) [22]. Surprisingly, the body soft tissues properties have almost no influence on the disk pressure variation. But this model is not suitable for a realistic description of both belt designs and body shapes.

The purpose of this paper is to develop a method to estimate the mechanical efficiency of a belt at the design stage through a mechanical model. The model should be run easily on the real body shape of any patient and should describe the belt placement and locking; it should be quick, easy to handle and use reasonable computer resources. We have chosen to use semi-analytical methods to estimate belt properties, belt stretch, and the pressure applied on the trunk, allowing us to investigate belt efficiency variations with sex, age and varying body morphology.

## Material and methods

**Fig 1:**
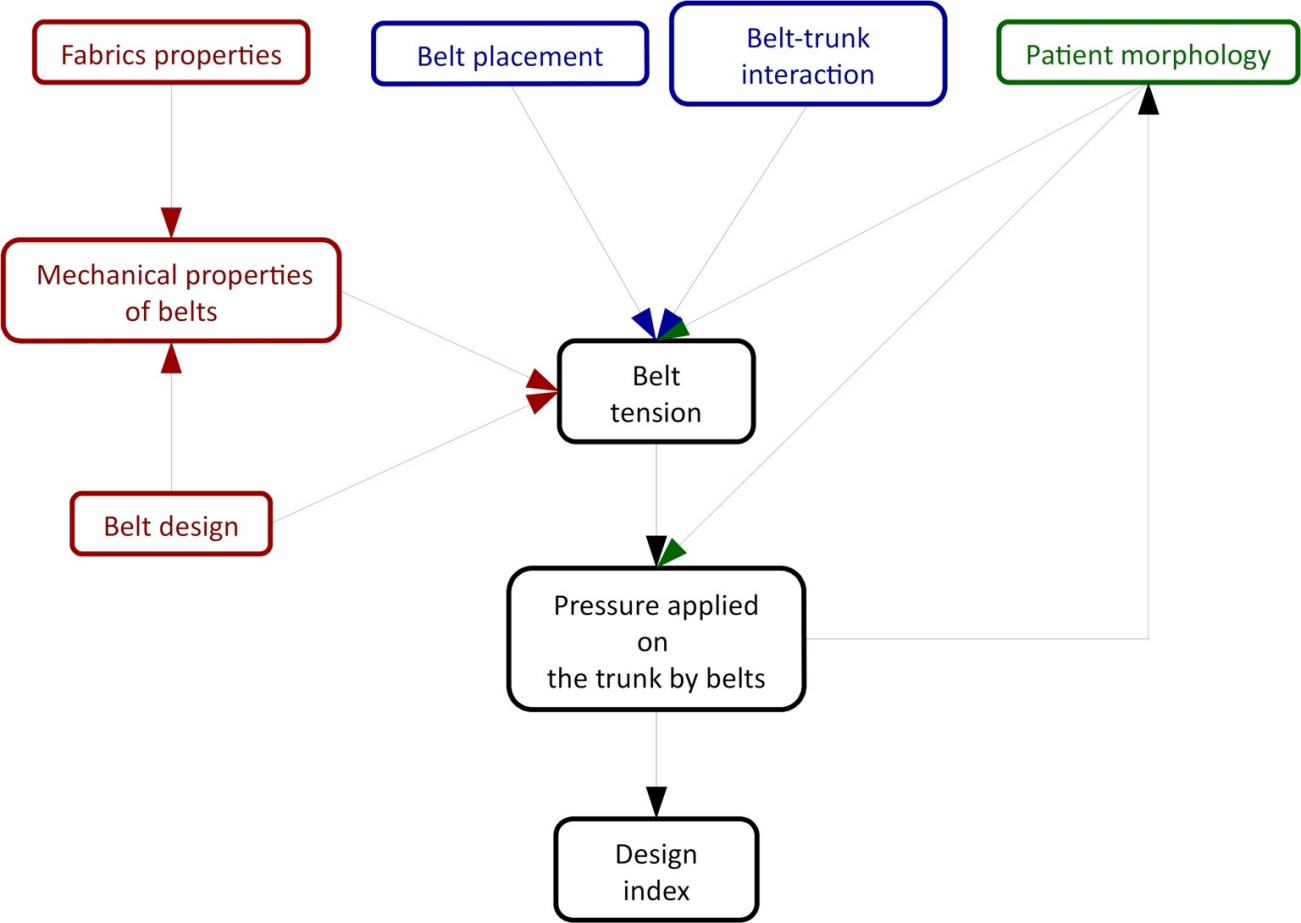
Methodology used to evaluate lumbar belts.

The global methodology of the study is summarised in Fig 1. Following the law of Laplace, the pressure applied by the lumbar belt on the trunk is proportional to the belt tension and the trunk curvature. Belt tension is the product of its stretch and its stiffness. The belt stretch is obtained by comparing the belt length and the trunk diameter. Belt mechanical properties will be estimated from the stiffness of the component materials and the chosen belt architecture. Patient morphology is measured using a linear triangulation scanner (Orten, France) and derived twice to obtain local curvatures for each specific patient. Typically, one patient shape measurement is made of 100,000 measuring points.

Previous research showed certain limitations to the law of Laplace [23]. First, the pressure applied on a deformable structure changes its shape, and therefore has an effect on the curvature radius and on the pressure produced. Therefore, this phenomenon was added to our model by calculating iteratively the pressure using the up-to-date deformed shape. Secondly, the belt has a tendency to stick to the body, and the law of Laplace can be modified to render the variations of tension induced by the adhesion. All these considerations will be explained extensively below.

### Lumbar belts

**Fig 2:**
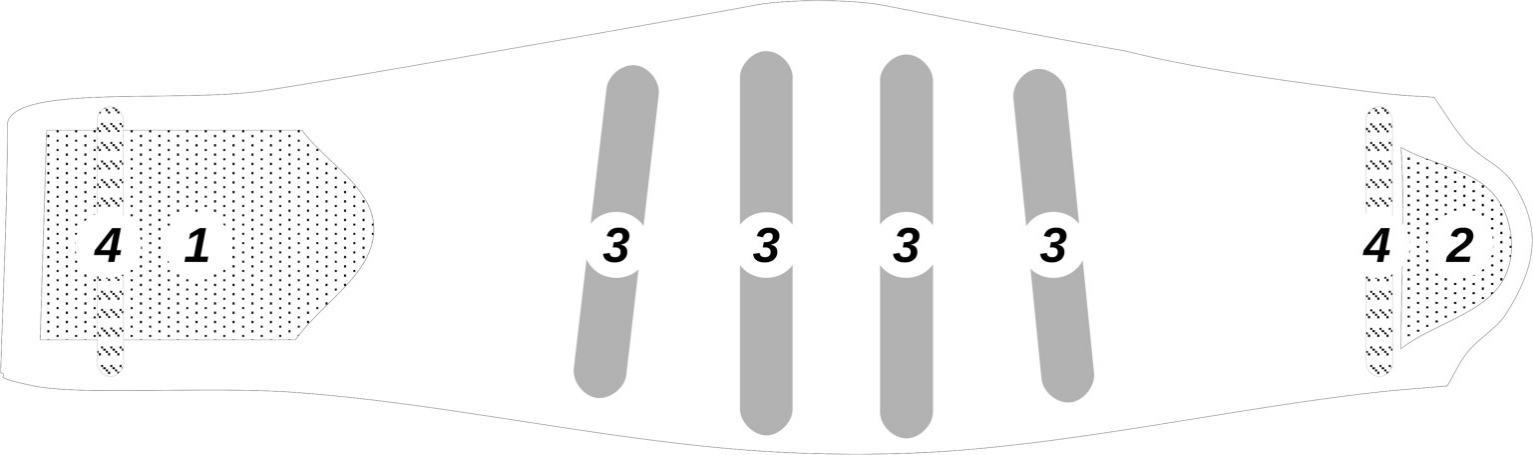
Schematic lumbar belt according to the French Healthcare system recommendation. 1 and 2: fastening system, 3: rigid whalebones, 4: soft whalebones.

In this study, two different lumbar belts models from Thuasne, Levallois-Perret, France have been evaluated. They will be denoted hereafter Belt A and Belt B. They have the same architecture: four rigid anatomic whalebones in the dorsal element, and two soft whalebones in the abdominal, corresponding to the French healthcare system recommendations [24] and can be considered as representative products on this market (see Fig 2). Mechanical behaviour of these belts is commonly described as a compression on the abdomen creating increasing abdominal pressure that provokes a change in spine posture. A description of fabrics constituting lumbar belts are given in Table 1. Manufacturer indications for the two lumbar belts are given in Table 2.

**Table 1:** Description of the belt fabrics studied.

Fabrics	Description	Composition	mass per unit area (g.m^-2^)	Specimen size
1	Twill	55% Polyester and Viscose, 23% Polyester, 22% Elastanne	725	width: 50 mm, length: 100 mm
2	Double face plain weave	75% Polyamide, 14% Polyester, 11% Elastanne	465	width: 50 mm, length: 100 mm
3	Coated plain weave	80% PVC 12% Polyester 8% Cotton	860	width: 50 mm, length: 200 mm
4	Plain weave	99.5% Polyester, 0.5% Polyamide	220	width: 35 mm, length: 200 mm
5	PU laminated brushed knit	53% Polyurethane, 47% Polyamide	200	width: 50 mm, length: 200 mm

**Table 2:** Manufacturer indications for the two lumbar belts analysed in the study.

Lumbar belts	Manufacturer indications	Composed by
Belt A	Static muscle deficiencies, osteoporosis, irritation of the sacroiliac joints and degenerative lesions	fabrics 1 and 3
Belt B	Low back pain with or without radicular pain in acute, subacute or chronic phase, the maintaining after lumbosacral pelvic surgery, connective tissue diseases of fibromyalgia.	fabrics 2, 4 and 5

### Cohort of patients

15 patients with chronic low back pain were followed in a complete clinical study. The measurement protocol was approved by the local Ethical Committee (CPP Sud-Est I: 2013-A01782-43). The 15 patients were recruited at the Physical Medicine and Rehabilitation Department of Saint-Etienne University Hospital. All the patients gave their informed consent. The panel was composed of 8 women and 7 men, from 26 to 56 years old. The BMI varied from 21 to 38, with 6 “healthy weight”, 8 “overweight” and 1 “severely obese” individuals. Patient characteristics are given Table 3. An Anderson-Darling normality test has been used to detect possible outliers; even if the BMI for patient #15 seems far off, this patient cannot be rejected.

**Table 3:** Patient characteristics. ^(*)^ healthy weight, ^(**)^ overweight, ^(***)^ severely obese.

Patient nb	sex	Age (years)	Height (m)	Weight (kg)	BMI (kg.m^-2^)
#1	F	49	1.62	55	20.96^(*)^
#2	F	27	1.61	55	21.22^(*)^
#3	F	49	1.70	62	21.45^(*)^
#4	F	26	1.72	66	22.31^(*)^
#5	M	35	1.77	72	22.98^(*)^
#6	M	45	1.76	73	23.56^(*)^
#7	M	36	1.80	84	25.92^(**)^
#8	M	56	1.68	74	26.22^(**)^
#9	F	47	1.68	74	26.22^(**)^
#10	F	46	1.69	75	26.26^(**)^
#11	F	31	1.75	81	26.45^(**)^
#12	M	46	1.76	90	29.05^(**)^
#13	F	37	1.67	85	30.48^(**)^
#14	M	50	1.82	101	30.49^(**)^
#15	M	29	1.74	115	37.98^(***)^
	min	26	1.61	55	21
	max	56	1.82	115	38

For each patient, the trunk shape was recorded using a 3D line scanner (Orten, France), resulting in a non-uniform cloud of points. The results were equally sampled into a cylindrical frame of reference; radii were slightly filtered using an outlier detection algorithm and a Gaussian low-pass filter, and curvature along the direction of the belt (the angular one) was extracted from a local 2^nd^ order polynomial fit.

### Belt placement

In order to fit with clinical practice, belts were positioned in the longitudinal axis so that the centre of support region laid over the centre of the lumbar spine curvature (L3/L4 vertebra). This way, belts partially covered the iliac crest and could lie on the 11^th^ and 12^th^ ribs. A preliminary study perturbing this placement (+/- 12 mm in the horizontal or vertical direction) showed that the belt positioning had only a negligible effect compared to other variables (belt model, age, BMI, belt locking …). Therefore, no further investigation of the belt position was performed.

Even though the products were designed to be worn directly on the skin, patients sometimes wear their belts upon an under garment. Consequently, an under garment/skin and an under garment/belt contacts should exist. In fact, textile/textile contact has very high friction coefficient and stick in most situations (see for example [25]), so the only contact system that should change in this case is the garment/skin one, yet another textile/skin contact. No argument supports a difference between under garment and lumbar belt in their contact behaviour; in the following, contact condition will therefore refer to skin/textile contact without precision on the nature of this textile.

#### Mechanical characterization of fabrics and belts

The tensile test of fabrics was conducted according to the European standard EN ISO 13934–1:1999 with an Instron 3343 Universal testing machine. Five samples were used in both weft and warp direction for each of the five fabrics. Sample size is given in Table 1. Fabric 4 was tested only in weft direction because this fabric is a 35 mm wide ribbon. According to the above standard, experimental speed was 100 mm/min. A preload of 5 N was applied, and the maximal load was set at 500 N.

Tests performed with the five samples per direction per fabric were not identical. The first sample was loaded three times in traction to determine the maximum elongation. For the second sample, load was applied three times in traction, and then released at the same rate. Repetition was performed to determine if the mechanical behaviour of the fabric changed after a number of loadings. For the three last samples, the load was applied twice in traction and then released at the same rate to check the hysteresis behaviour of the fabric. Only the second loading was analysed.

True strain ε in the direction of the tensile test was extracted from movable jaw displacement of the tensile tester. During all experiments, pictures were taken every second to follow the evolution of the true strain ε_t_ in the transverse direction. Poisson’s ratio ν was subsequently extracted from these images. The load was measured using a 500 N load cell; because fabric thickness is difficult to define and to measure, tensile force per unit width T (in N/mm) was used instead of stress.

**Fig 3:**
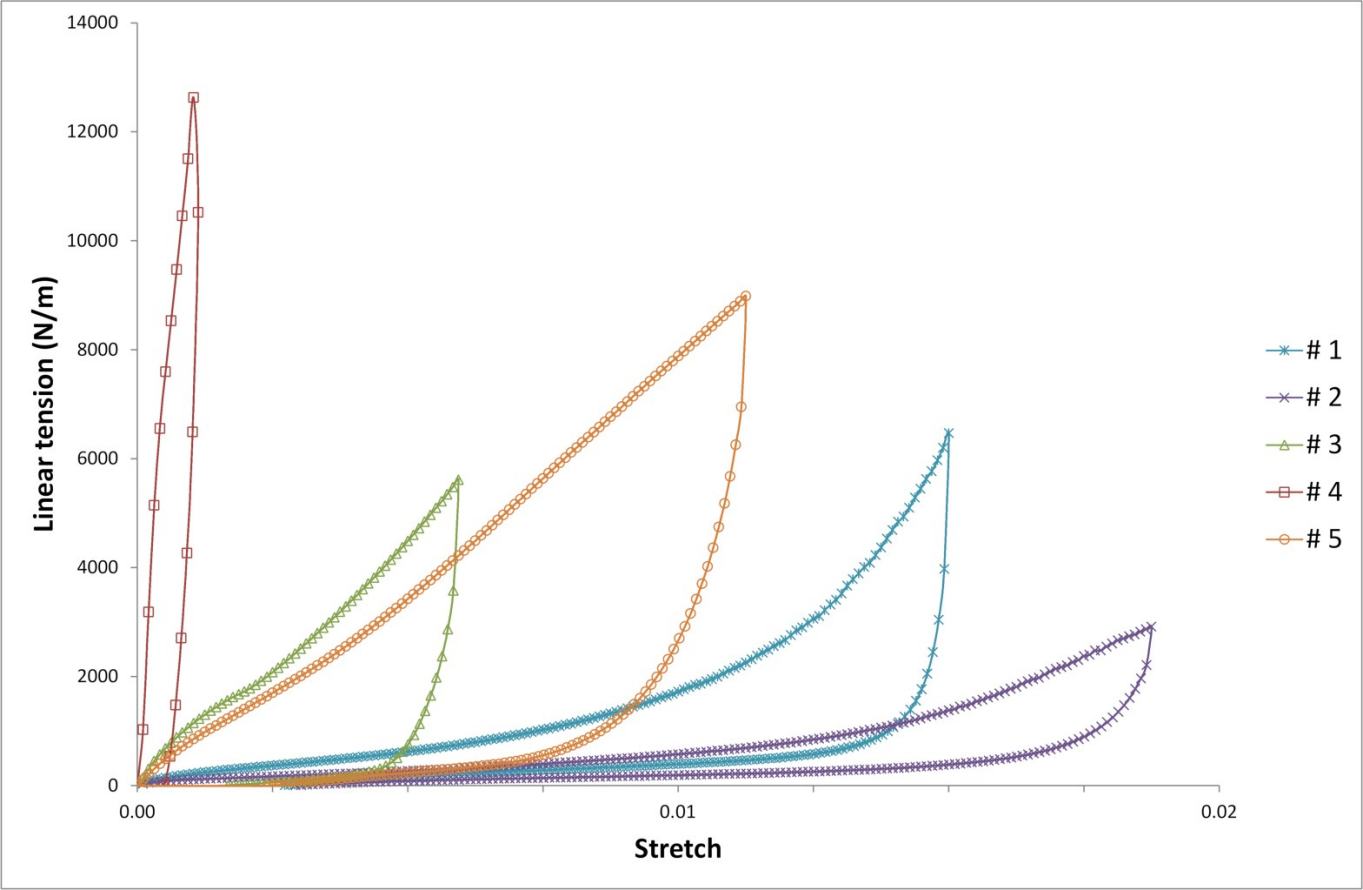
Tensile force per unit width vs stretch curve for the five studied fabrics (in warp direction).

The typical tensile force per unit width vs. strain curve obtained in this study has two phases: a rising phase during traction and a descending phase when unloading. Typically, the belts are stretched and locked onto the body when relaxing the load therefore we decided to define the stiffness as the tangent’s slope at the beginning of the unloading part of tensile force per unit width vs. strain curve. Illustration of this non-linear behaviour is given in Fig 3.

**Fig 4:**
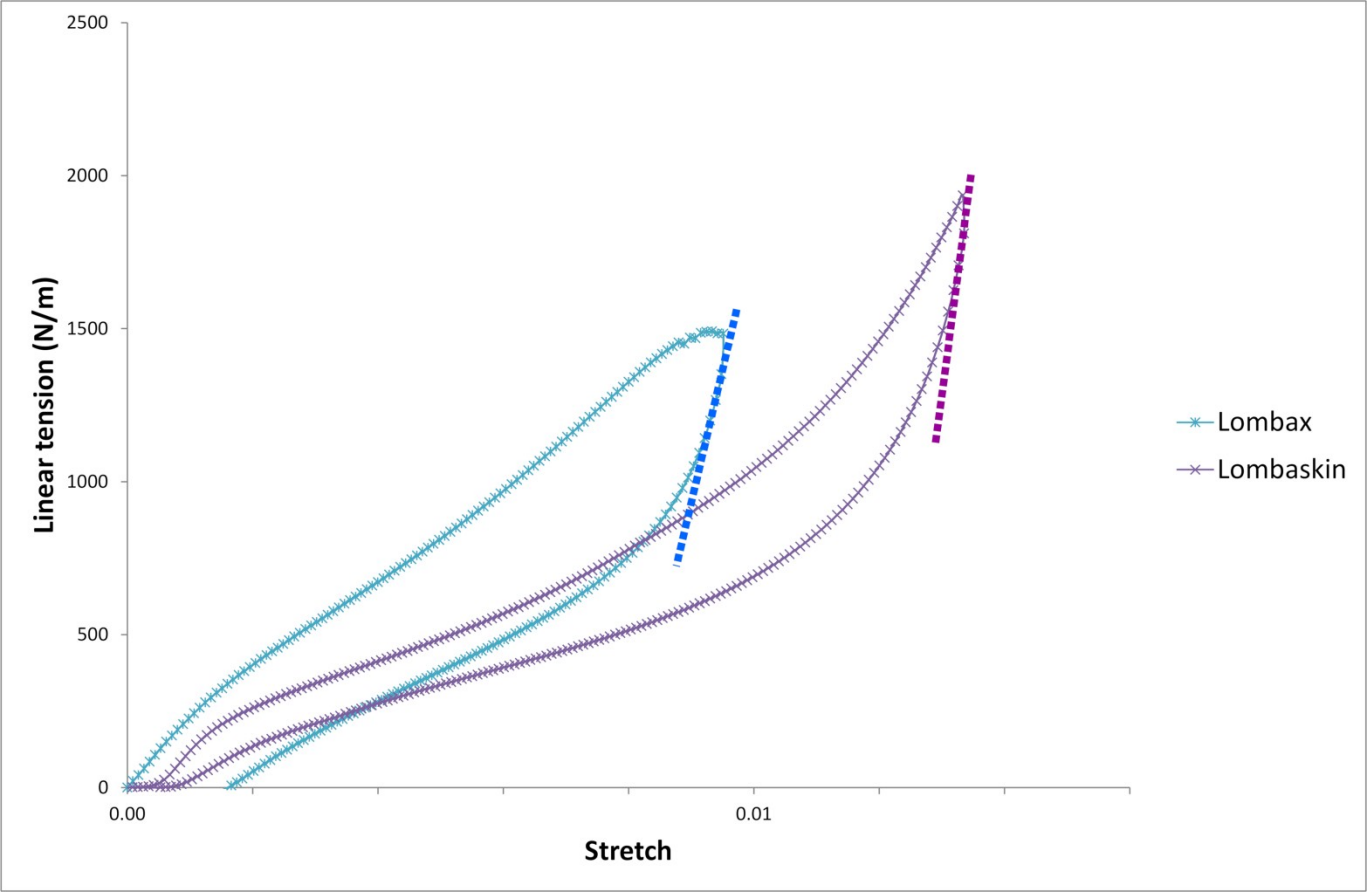
Force-displacement curves for the two studied lumbar belts.

Lumbar belts were tested in a similar way to the fabrics. They were fixed on the Instrom 3343 universal testing machine using adapted fixtures. Three samples per belt types were tested. All loads were applied in traction then released three times to check plasticity, hysteresis and fatigue behaviour. The typical force-displacement curve for lumbar belt is the same as for linear tension vs strain curve of fabric, with a substantial hysteresis. Being consistent with the definition of stiffness for the fabrics, stiffness of a lumbar belt is defined as the tangent’s slope at the beginning of the unloading part of the force-displacement curve (dashed lines on Fig 4).

### Mechanical model of lumbar belts

**Fig 5:**
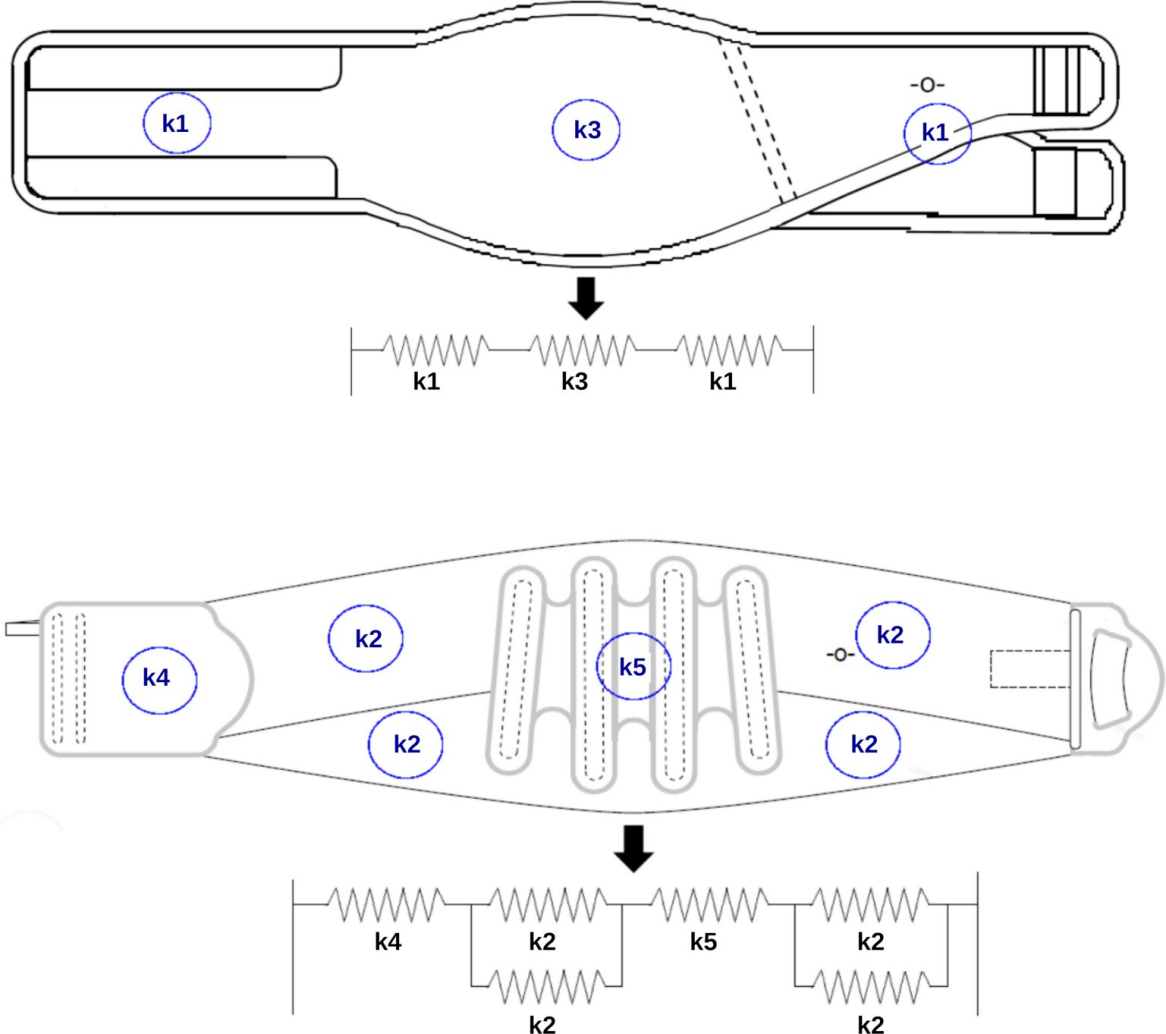
Model with springs of: a. Belt A, b. Belt B.

To determine the relationship between the mechanical behaviour of fabrics and the mechanical behaviour of the corresponding lumbar belts, a simple mechanical model is proposed. Lumbar belts are fabric assemblies which differ widely depending on their structure. Each fabric is modelled by a spring with a given stiffness. The models that we developed for the two belts are shown on Fig 5, in which stiffness k_1_ is the stiffness of fabric 1; k_2_ the stiffness of fabric 2 and so forth. Equivalent stiffness k_eq_ is the sum of fabric stiffnesses when springs are parallel, and the inverse of the sum of fabric compliances when springs are serial.

For example, equivalent stiffness of Belt B is given by the Eq 1:

$$k_{eq}=\frac{2\times k_{2}^{2}\times k_{3}\times k_{5}}{2\times k_{2}^{2}\times k_{5}+k_{2}\times k_{3}\times k_{5}+2\times k_{2}^{2}\times k_{3}+k_{2}\times k_{3}\times k_{5}}$$ (1)

### Interface pressure calculation

According to the law of Laplace, the pressure *p* applied on the trunk by a lumbar belt is linked to the radius of curvature *R* of the trunk’s tensile force per unit width *T* of fabrics contained in lumbar belt as following [26, 27]:

$$p=\frac{T_{1}}{R_{1}}+\frac{T_{2}}{R_{2}},\;T_{1,}T_{2}\geq 0,\;R_{1,}R_{2}>0$$ (2)

Direction 1 is the direction of the belt extension and direction 2 is perpendicular to this direction. The pressure estimated by Eq (2) is valid only for convex surfaces. If the curvature is concave, then the pressure is set to zero, as the belt would not be in contact with the trunk.

The law of Laplace is based on strong assumptions: it is assumed that the two directions of traction correspond to the two main curvature radii; it does not consider volumetric effects in the belt, and assumes the belt is sliding on the trunk without any friction.

Curvature radius R is given by Eq 3:

$$R=\frac{1}{\gamma }=\frac{{\left(1+y^{\prime }{\left(x\right)}^{2}\right)}^{\frac{3}{2}}}{y^{\prime \prime }(x)}$$ (3)

Where γ is the curvature and (*x*, *y*, *z*), the Cartesian coordinates of any point *M* on the surface of the trunk. These coordinates are obtained by the 3D line scanner.

### Tension assessment with or without sticking

The strain as well as curvature should be defined locally. Here, only the belt variation along the warp direction is considered. This can be assumed to be a negligible shear strain effect, which is reasonable in most fabric weaving. Strain in warp direction is then projected in the longitudinal and transverse direction, giving respectively ε_1_ and ε_2_. Another important assumption is that belt placement doesn't reorient the textile tows.

Strain estimation is based on the local mechanical equilibrium in presence of adhesion. If τ represents the local interface shear, sticking conditions can classically be written as:

$$\left|\frac{\tau }{p}\right|\leq a$$ (4)

Then, using *s* as the local coordinate, it is easy to write the local mechanical equilibrium of an infinitesimal element under tension as:

$$p(s)=\frac{T(s)}{R(s)}$$ (5)

$$\frac{\delta T}{\delta s}(s)+\frac{\xi a}{R(s)}\times T(s)=0$$ (6)

Eq (4) defines two limiting situations, 0 ≤ |*τ*| ≤ *a*|*p*|. The lower limit with no interface shear is equivalent to a pure sliding situation, and the upper limit |*τ*| = *a*|*p*| corresponds to a maximization of sticking effect. The true situation should lie between these two limits. The parameter ξ represents this situation in Eq (6), but also the friction being always in the opposite direction of the movement. The two limiting situations will be explored further.

### Shape remodeling

Because body tissues are soft, they deform under the pressure applied by the belt. In many cases, a finite element model could be used [22]. The main advantage of this approach is the possibility of describing mechanically the inner organs (liver, intestine, bones …), however the knowledge of inner organ properties and geometry is not easy to obtain in the current medical practice. Moreover, the finite element modelling is time consuming. Here, our aim was to get a better pressure map by correcting the curvature radii, without using a detailed description of the inner part of the abdomen. In order to achieve a simple and accurate correction, we assumed a homogeneous elastic linear model for the abdomen. This assumption is supported by a sensitivity analysis presented recently showing that the belt efficiency poorly depends on the abdomen properties [28]. As a drawback, it is impossible to render the effect of bone on the pressure generation. This assumption is realistic where belts are pressing the 11^th^ or 12^th^ ribs–the floating ribs, but it could give unrealistic pressure maps near iliac crests, especially for slim patients.

In 1929, Love reported an analytical solution for the deflection of a semi-infinite body submitted to a rectangular punch [29]. At a point *M*(*x*, *y*) on the free surface of a semi-infinite body submitted to a pressure *p* over a rectangular area, the displacement *u*_*z*_(*x*, *y*) is given by:

$$\begin{array}{l} \frac{\pi E_{b}}{1-\nu_{b}^{2}}\frac{u_{z}(x,y)}{p}=(x+a)ln[\frac{(y+b)+{\left\{{\left(y+b\right)}^{2}+{\left(x+a\right)}^{2}\right\}}^{1/2}}{(y-b)+{\left\{{\left(y-b\right)}^{2}+{\left(x+a\right)}^{2}\right\}}^{1/2}}]+\\ \;(y+b)ln[\frac{(x+a)+{\left\{{\left(y+b\right)}^{2}+{\left(x+a\right)}^{2}\right\}}^{1/2}}{(x-a)+{\left\{{\left(y+b\right)}^{2}+{\left(x-a\right)}^{2}\right\}}^{1/2}}]+\\ \;(x-a)ln[\frac{(y-b)+{\left\{{\left(y-b\right)}^{2}+{\left(x-a\right)}^{2}\right\}}^{1/2}}{(y+b)+{\left\{{\left(y+b\right)}^{2}+{\left(x-a\right)}^{2}\right\}}^{1/2}}]+\\ \;\left(y-b)ln[\frac{(x-a)+{\left\{{\left(y-b\right)}^{2}+{\left(x-a\right)}^{2}\right\}}^{1/2}}{(x+a)+{\left\{{\left(y-b\right)}^{2}+{\left(x+a\right)}^{2}\right\}}^{1/2}}\right] \end{array}$$ (7)

with

- the pressure p as $\begin{array}{c} |x|<a\wedge |y|<b,\;p(x,y)=p\\ |x|\geq a\vee |y|\geq b,\;p(x,y)=0 \end{array}$
- *E*_*b*_ and ν_*b*_ the Young’s modulus and Poisson ratio of the semi-infinite body.

Then, for a distributed pressure, applying the superposition principle gives the displacement of the point *M*(*x*, *y*) as the sum of all the individual normal displacements related to each pressure component. Here, it is worth noting that the surface is neither flat nor semi-infinite. If the pressure is set over a very small area *a*×*b* corresponding to the sampling of pressure maps, the curvature radius is orders of magnitude higher than the perturbation length–typically several times *a* or *b*. In such a situation, curvature of the surface is locally negligible. In the same way, trunk dimension is infinite compared with the perturbation length, and the semi-infinite assumption is justified.

Now, even in the small perturbation context, the integral of all the deflection caused by each individual pressure contribution can cause a global variation in the curvature radius, and in turn a change in the applied pressure itself, as stated by the law of Laplace (Eq 2). The mechanical equilibrium is achieved when the pressure used as an input of the deformation scheme and the pressure calculated using the law of Laplace with the curvatures in the deformed state are the same. Here, an explicit incremental procedure has been implemented. This method is suitable to calculate the actual pressure field while respecting step by step the conditions of the superimposition principle.

### Mechanical analysis

Pressure applied on the trunk can be described using the screw theory. For each section, a reduction force vector is calculated by integrating the pressure over the corresponding surface. Reduction force is applied at the centre of force for each section. Then, a global torque is calculated. The point where the torque is reduced should correspond to a specific location on the spine. Here, because the methodology gives only external information, torque is calculated at the centre of the lumbar curvature on the body surface, which we define as the most anterior lumbar vertebral body. It is as close as possible to the L3 or L4 vertebrae. The transversal component of the torque is of high interest: depending on the sign, its effect decreases the lumbar lordosis angle when the value is positive or increases it if negative.

### Statistical methods

Sensitivity to qualitative parameters (sex, belt model, belt locking) was assessed using paired (belt model, belt locking) or unpaired (sex) difference tests. When the normality hypothesis was verified, Student's t test was used. Wilcoxon or Mann-Witney non-parametric tests were applied to non-compliant data sets. Quantitative parameters (BMI, coefficient of adhesion) were analysed with a linear regression model. Significance on sensitivity parameters is calculated using a Student-t test. The risk factor for all statistical tests was set to 5%.

## Results

### Equivalent belt modeling

#### Fabrics characterization

For all fabrics, mechanical behaviour is a little bit different during the first loading of the sample, but the following repetitions show similar behaviour. These results show that fabrics of lumbar belts must be loaded once before having a stable mechanical behaviour. Fig 4 shows tensile force per unit width vs stretch curve for the five studied fabrics. As explained earlier, stiffness is taken at the beginning of the unloading part of the curve. Stiffness and Poisson’s ratio for all studied fabrics are given in Table 4.

**Table 4:** Stiffness and Poisson’s ratio of studied fabrics.

Fabrics	In warp direction		In weft direction	
	Stiffness (N.m^-1^)	Poisson’s ratio	Stiffness (N.m^-1^)	Poisson’s ratio
1	39 226	-0.08	38 948	-0.32
2	16 611	-0.03	52 772	-0.35
3	41 421	0.52	32 321	0.45
4	101 433	0.30	—	—
5	51 429	0.46	57470	0.55

#### Stiffness of lumbar belts

Force-displacement curves for both lumbar belts are represented in Fig 5. As for the fabrics, the belt force-displacement curves are non-linear and show hysteresis. Due to the mechanical model of lumbar belts, the equivalent stiffness at the beginning of unloading was calculated for each belt type. The results, compared to the stiffness obtained by tensile test, are given in Table 5. The relative error between the equivalent stiffness obtained from the fabric properties and the actual stiffness from a direct tensile test remain reasonable, less than 8%.

**Table 5:** Stiffness and equivalent stiffness of lumbar belts.

Lumbar belts	Stiffness (kN.m^-1^)	Equivalent stiffness (kN.m^-1^)	Error (%)
Belt A	12.6	12.1	3.29
Belt B	12.4	11.4	7.68

### Trunk-Belt interactions

15 patient shapes were processed, but only 11 gave results, the body shape quality being insufficient for the other 4. The two selected belts were placed numerically, in order to ensure a nominal 20% stretch before application on the body, as it would be in an ideal case according to the manufacturer's recommendation. Overall, 96 different situations have been generated and analysed.

#### Belt strain and pressure maps

**Fig 6:**
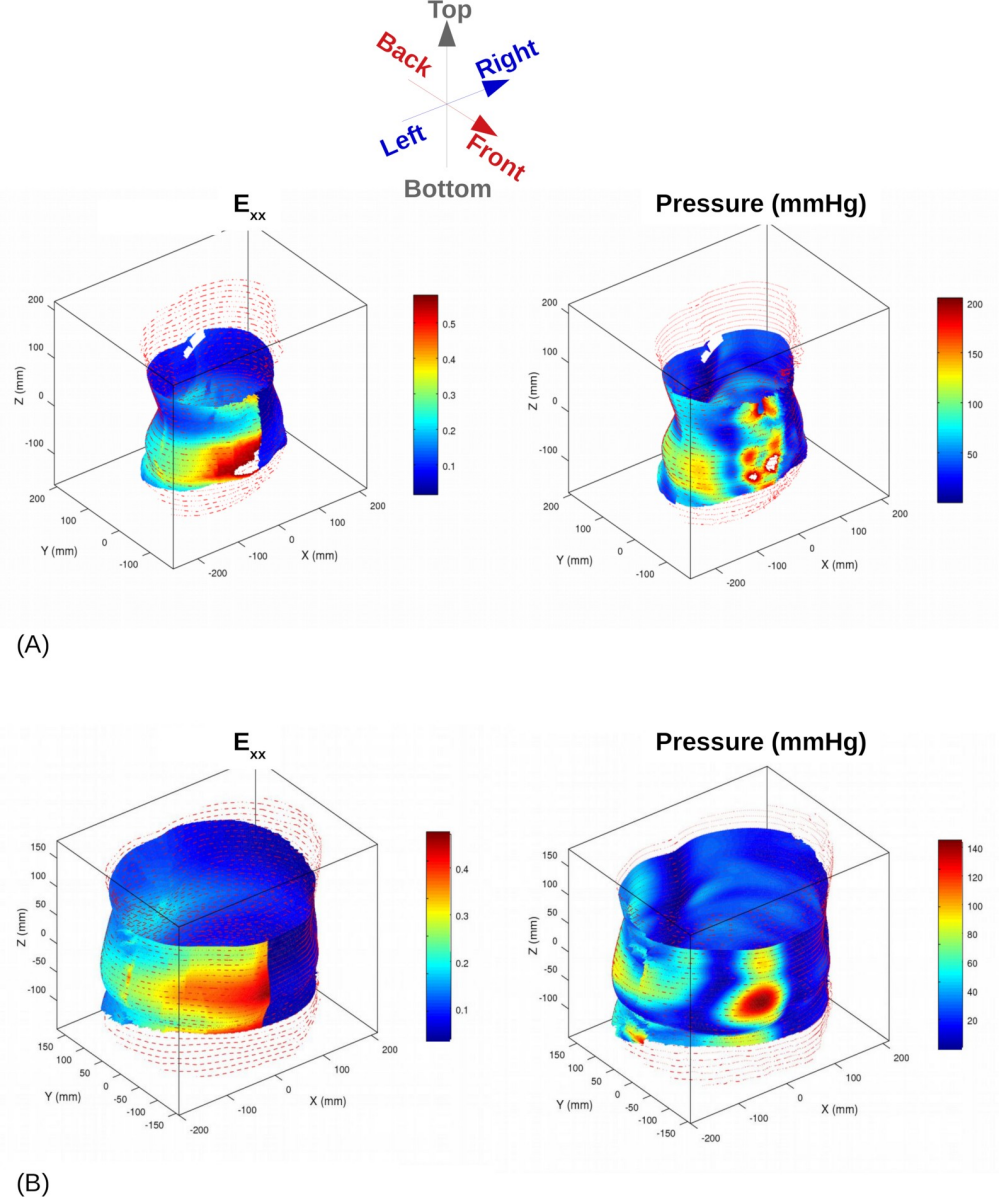
Belt strain and pressure obtained numerically for (A) 27 y.o. woman, BMI = 21.2 kg/m^2^ and (B) 46 y.o. man, BMI = 29.0 kg/m^2^. (Belt A case, CoA = 0.3, asymmetric locking).

Belt strain distribution is illustrated on Fig 6 (left side) for 2 representative patients. It is worth noting that the strain follows the global body shape, and is maximal on the chest, decreasing rapidly on the abdomen. The corresponding pressures are presented on the right. The pressure field is much more dependent on the morphology, which varies in this study with the patient's sex and BMI. Pressure induces a moderate body compression, from 2 to 5 mm depending on the patient; this change induces a decrease in the belt stretch.

**Fig 7:**
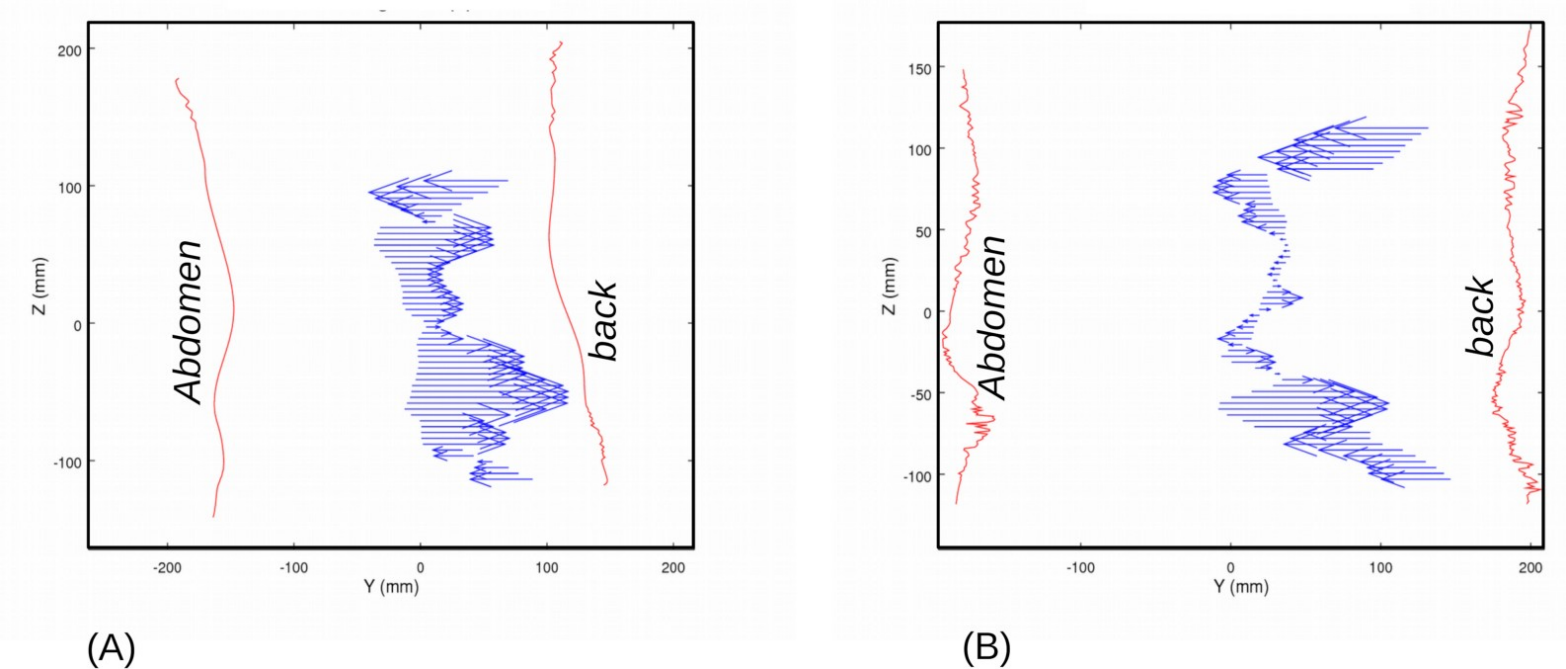
Resulting forces per cross section–sagittal view for (A) 27 y.o. woman, BMI = 21.2 kg/m^2^ and (B) 46 y.o. man, BMI = 29.0 kg/m^2^. (Belt A case, CoA = 0.3, asymmetric locking).

Additionally, resulting force vectors can be calculated as a series of slices in the transverse plane (Fig 7). The force vector is more pronounced in the antero-posterior axis, with a direction that could change at both edges of the belt, but the main result lies in the centre of force location. The centre of force location calculated for each slice is near the vertebrae on the two ends of the belt, and close to the centre of the trunk between the top and bottom of the support. This distribution is an indication of the presence of a bending moment that should be seen as a measure of the belt efficiency.

#### Belt effect

**Fig 8:**
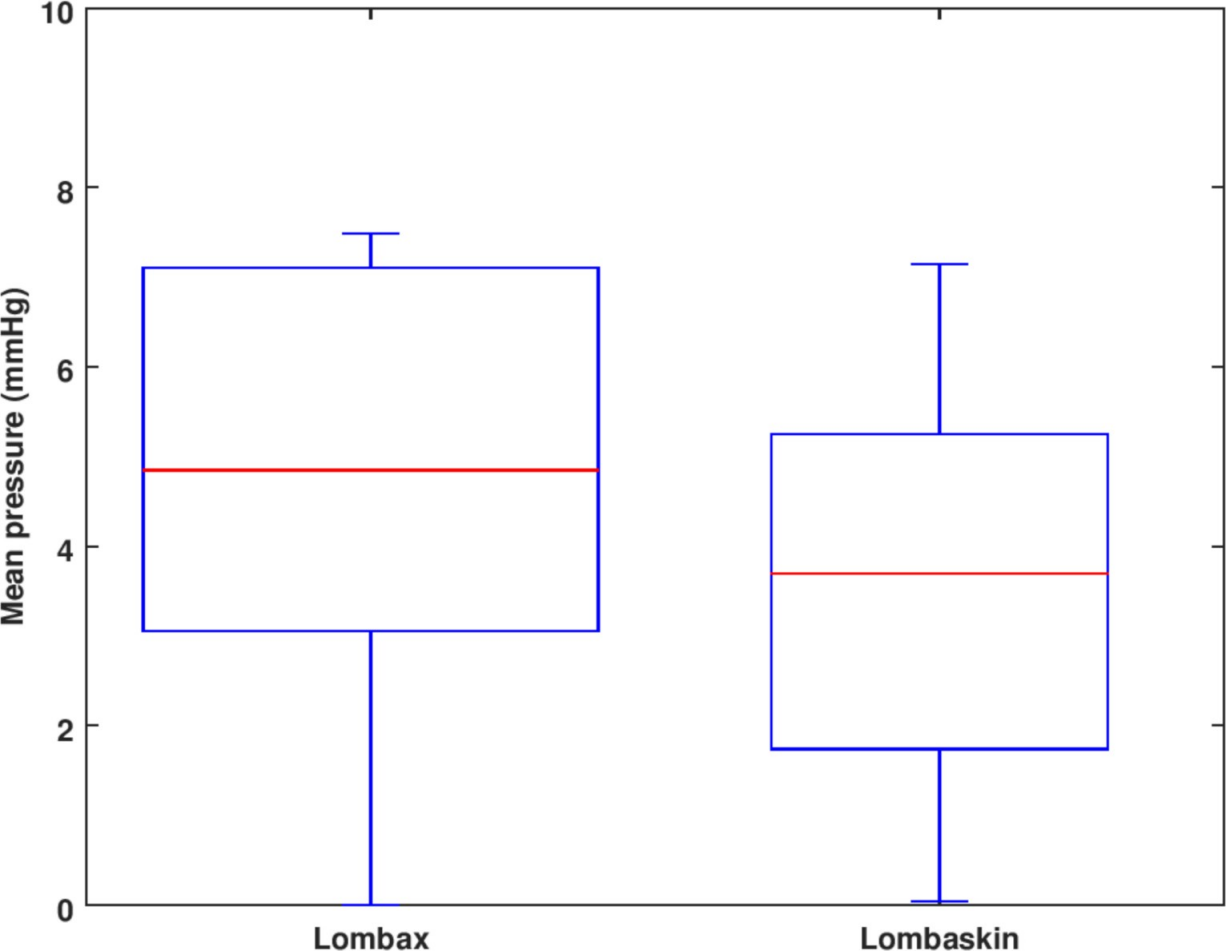
Mean pressure for the two belts.

In normal clinical practice, a lumbar belt is prescribed, but no information is provided on the belt choice. Yet the characteristics of the medical device itself are important in terms of efficiency and comfort. In our in-silico clinical study, tests on both the bending moment and the mean pressure failed to achieve normality, and a Wilcoxon paired test had to be used. It was impossible to conclude on a significant difference between the two belts for the bending moment, but there was a statistically significant difference between mean pressures (Fig 8).

#### Sex effect

A Mann-Witney U-test found no significant difference between sexes, even though qualitatively it seems that pressure is applied in a different way in the two populations. For patients with normal BMI, the pressure is maximal on male abdomens, whereas in women it is maximal on the back, at the beginning of glutei muscles. In both cases, pressure on the back is very limited, with a zero value along the spine and a small contribution on the longissimus thoracis muscles. For subjects with higher BMI, pressure is more homogeneous on the abdomen and on the back.

#### Belt-body sticking effects on the pressure generation

The coefficient of adhesion (CoA) between skin and textile varies in a wide range, from 0.1 to 0.5, depending on the skin contact pressure applied, skin hydration, moisture, thickness or patient age [30]. If adhesion occurs, one key question lies in the kinematics of the belt locking. Two representative situations were considered:

- Asymmetric locking: patients maintain the belt with their left hand while stretching and locking it with the right hand.
- Symmetric locking: patients stretch the belt symmetrically with both hands and close it on the abdomen.

**Fig 9:**
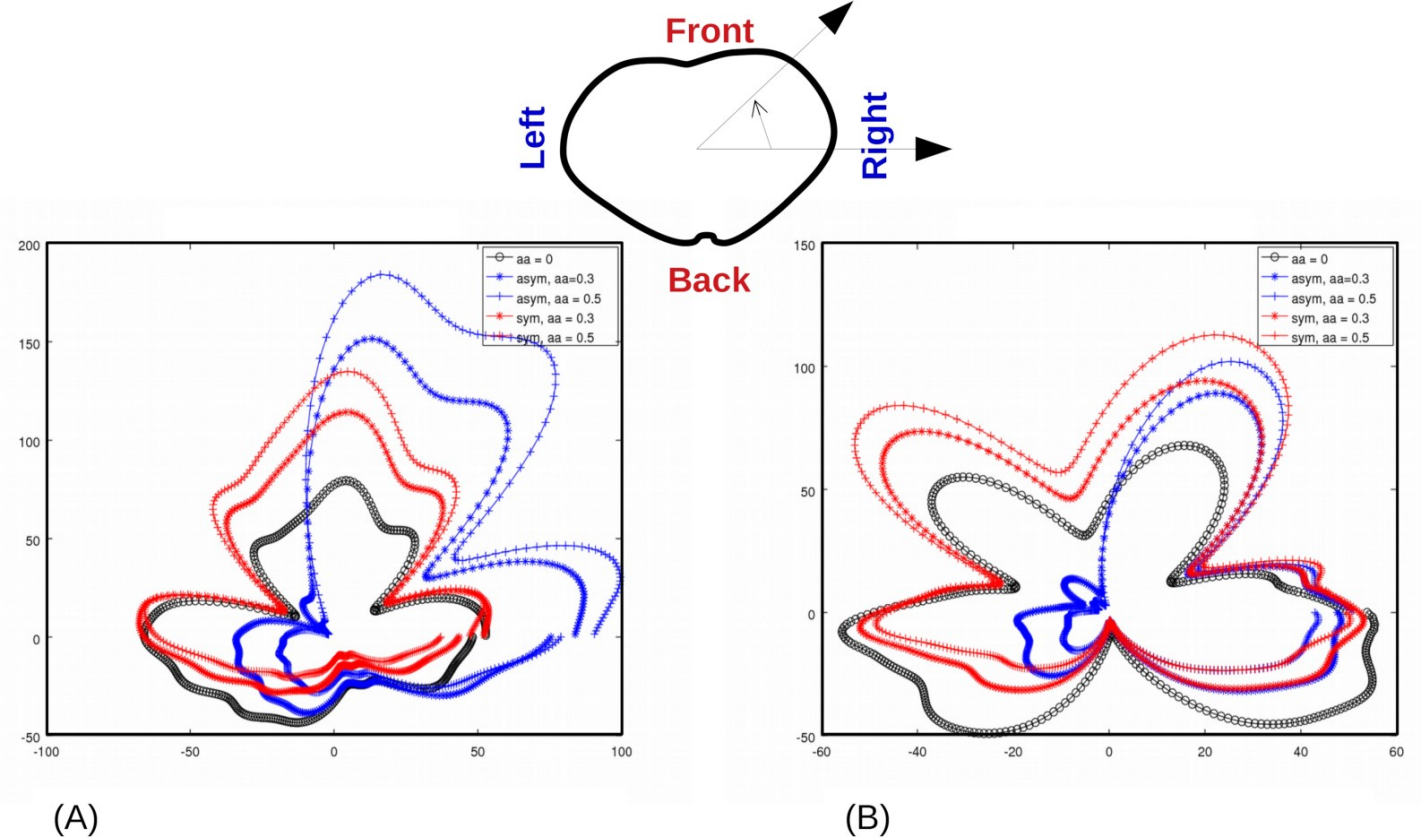
Mean pressure around the trunk depending on belt locking strategy and CoA (Belt A). Belt closure is at 90°, and patient’s back is centred at 270°. Illustrations are given for (A) 27 y.o. woman, BMI = 21.2 kg/m^2^ and (B) 46 y.o. man, BMI = 29.0 kg/m^2^.

**Fig 10:**
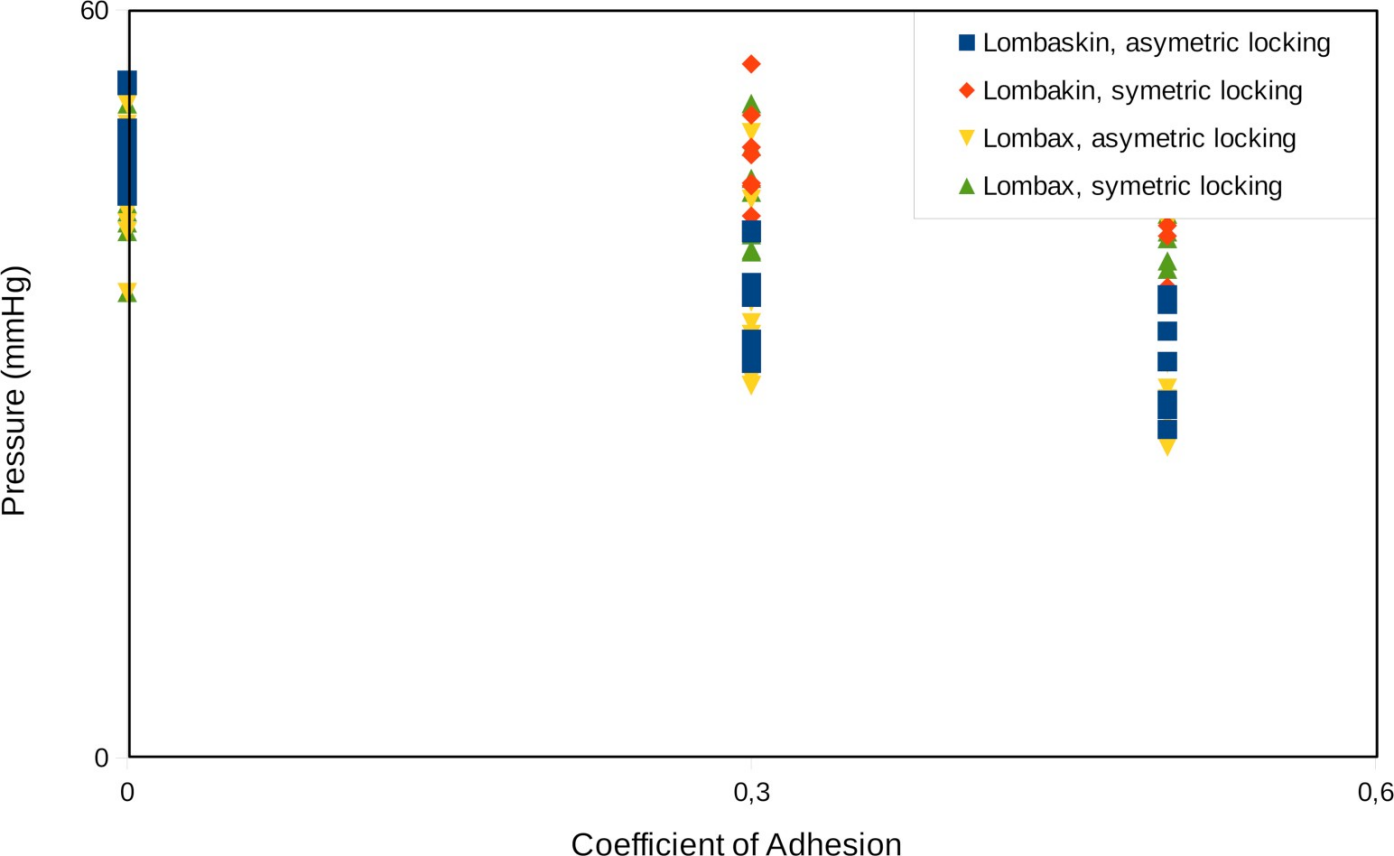
Mean pressure evolution with coefficient of adhesion.

Fig 9 reports the pressure generation around the patient’s trunk when symmetric or asymmetric locking for a friction coefficient set to 0.3 and 0.5, as well as results obtained in absence of sticking effects. Qualitatively, sticking effect clearly changes the pressure distribution for all patients. In particular, the pressure on the back is decreased and pressure on the abdomen increased in presence of sticking. Moreover, the pressure distribution becomes dramatically asymmetric on the abdomen when locking is asymmetric. The way the belt is locked has a significant influence on the mean pressure for Belt B (respectively Belt A) with a 30% (respectively 40%) variation and influences the bending moment. A multivariate regression analysis was performed on the coefficient of adhesion and the BMI for each belt and each locking procedure. Pressure decreases when the coefficient of adhesion increases. Variation is statistically significant only for Belt B (Wilcoxon paired test) but the trend is the same for Belt A. Results are presented Fig 10.

#### BMI effects

**Fig 11:**
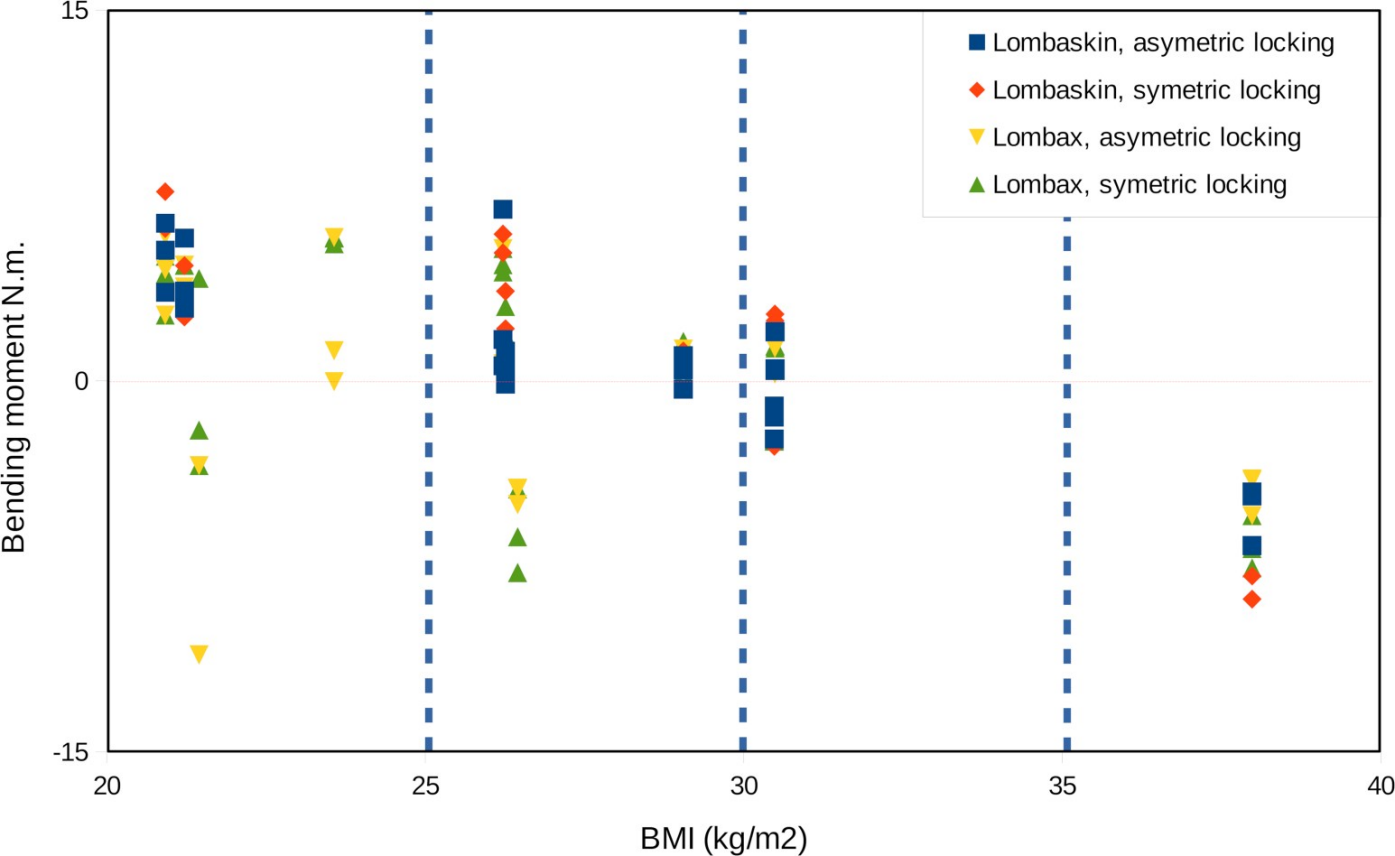
Bending moment vs BMI for all the patients and all the belts.

A regression analysis was performed for each belt and each locking procedure both on BMI and coefficient of adhesion. No coupling effects could be observed. BMI significantly influences the pressure and the bending moment. When the BMI increases, the mean pressure decreases with the bending moment. Despite inter-subject variability, Fig 11 shows that the bending moment is positive for healthy weights and tends towards zero for overweight patients and is negative for obese ones.

## Discussion

### Aims

We have developed a simple way of modelling lumbar belt mechanical effects on a trunk at the design stage. Because of this focus, robust mechanical methods have been chosen, in spite of the number of assumptions to be verified.

### Main findings

A first limited in silico pre-clinical trial shows the effect of two different belts on 15 different chronic low back pain patients, both men and women, from 26 to 56 y.o., with height from 1.61 m to 1.82 m, and weight from 55 kg to 115 kg (BMI ranging from 21 kg/m^2^ to 38 kg/m^2^). Two main indicators are extracted here: firstly, even though it is the driving mechanism of lumbar belts, the pressure they apply on the patient’s trunk can be seen as a source of discomfort. Secondly, a positive bending moment on the frontal axis corresponds to an effect of reduction of lumbar lordosis, and so, in the lumbar region, a decrease of the pressure in the distal part of the disk and an increase in the proximal one. Many reasons are invoked as a source of chronic low back pain, and many anatomical structures in question: apophyseal joints, intervertebral disk, spinal muscles, peri-articular structures, nerve compression, etc. [31]. Inadequate spine postures and mechanical solicitations are reported. So, besides other effects such as for example proprioceptive or antalgic effects, reduction of lumbar lordosis angle is researched as a therapeutic effect in order to decompress anatomical structure, to reduce constraints and nociceptor solicitations and relieve pain [32]. The best situation for such patient should be defined as the higher bending moment with the lower pressure. As our results show, pressure depends on the belt, its locking procedure, the Coefficient of Adhesion (CoA) and the Body Mass Index (BMI) whereas the bending moment only dependents on the BMI. The two belts tested show a statistically different bending moment, even though their stiffness is very similar. Therefore it can be concluded that the bending moment mainly depends on geometrical parameters, the body shape, and the belt design. As a key finding, it is demonstrated that pressure and bending moment do not depend on the same parameters, and that there is a possibility of optimizing a treatment regarding these two factors.

### Belt modelling

Belt stiffness is directly evaluated from its components and its design. The unidirectional mechanical model is sufficient to assess the stiffness of the belts with a mean error close to 6%. Here, the stiffnesses of the chosen belts are similar (12.4 and 12.6 kN.m^-1^), thus the results enhance the effect of textile arrangement. Our belt placement model is built on an extended law of Laplace, a simple relation linking the generated pressure on the body, the belt tension and the local curvature, but it is valid only if the belt is purely sliding on the surface and if the surface is rigid. A new development is proposed to overcome these two limitations. Firstly, the model includes the deformation of the trunk under pressure, using the Love approach for a semi-infinite linear elastic body. As a consequence, the body shape continuously changes when loading the belt. The pressure field must be adapted to the current geometry–and curvature radii. Here, an explicit scheme has been adopted; the loading step is defined to be small enough to give a stable pressure field. The maximum effective deformation is 5 mm, which validates the semi-infinite assumption on the soft parts of the body. Secondly, the paper presents a first attempt to take into account adhesion in the belt mechanics. This approach should be a valuable tool in the study of other compression or contention medical devices.

### Belt effect on lordosis

Cohort has been studied according to the Body Mass Index and the sex which refer to the patient morphology. Influence of the BMI both on pressure and on bending moment is of high practical interest. This change in moment implies that the reduction of lumbar lordosis depends on the BMI. Even if the conditions are quite different and making detailed comparison seems hazardous, these results show the same trends as in [33]. A consequence of Fig 11 should be that lumbar belts are not recommended for obese patients with hyperlordosis, because the belt moment tends to increase the lumbar lordosis angle. Therefore, some specific solutions should be designed for this population. The mechanical explanation to this lies in the pressure distribution. For “healthy weight” patients, a difference of pressure appears from front to back, with a maximum pressure on the anterior face. This difference disappears for higher BMI patients, with a pressure spread all around the trunk as it compresses mainly the fat tissues. These results question the classical explanation of the belts mechanical effect. The bending moment is related to the distribution of pressure around the body rather than to the pressure on the anterior face. Even though no statistical difference was found between men and women, this explanation outlines the geometrical effects and nonetheless suggests studying sex differences in more details.

### Sticking influence on the belt

By varying the Coefficient of Adhesion between 0 and 0.5, our results show that adhesion can dramatically change pressure distribution. The asymmetric locking procedure, coupled with adhesion, results in a high left/right pressure difference. As a consequence, it gives a parasitic bending moment along the anterior-posterior axis. Moreover, in the examples in Fig 9 the ratio of the pressure on the right side of the body to the mean pressure is 2.6 times higher than in the frictionless case. This could be a source of discomfort or even skin irritation. The back-to-front pressure distribution is also changed, with a ratio of the mean pressure on the back versus the abdomen increased up to 2.4 times than in the frictionless case. A better control of the adhesion might results in a change in the bending moment and a better efficiency of the belt but contact conditions (friction or adhesion) are difficult to characterize, because they depend on various physical parameters, including the pressure applied on the skin, and skin biophysical state (hydration, moisture …). In the literature, very few works provide skin/textile Coefficient of Adhesion, and the friction coefficient is commonly the variable of interest; typical values vary from 0.1 to 0.5. This first adhesion model clearly indicates that textile/skin contact is an issue in pressure generation and must be studied in more details in the future, for example by studying the textile reorganization with the patient movements.

### Validity of the mathematical models

This model can be compared to the clinical study recently conducted by R. Bonnaire on the same belts [21, 34]. The nominal belt stretch was the same as in this study (20%). Experimentally, the maximum pressure was 60 to 70 mmHg and a pressure close to zero in the lumbar back of the patients has been reported. Yet, this experimental pressure could be measured only on the lumbar back and on the lateral sides but not on the anterior side of the abdomen. Experiments showed that both strain and pressure were significantly different between the left and the right lateral sides of patients’ abdomens; the pressure on the right side is twice that of the left side. All the belts had an asymmetric locking system, thus, the adhesion effect could explain this difference. Numerical and clinical trends show good coherence in the pressure range, as well as in its distribution (zero-level on the back, a different pressure on the two sides of the abdomen). Still, this comparison is incomplete and more work is required for a complete validation of the proposed model.

### Limitations

Even if this model gives a new way to study lumbar belts effects, with clear results, some limitations must be recalled. Firstly, the material properties for the belt and the trunk are insufficient to describe belt behaviour after long wear, and other phenomena should be considered, such as viscoelaticity. Moreover, textile behaviour is strongly anisotropic and only one component of the stiffness tensor is used here. This is clearly justified in so far as the tension applied to the belt corresponds to textile axis (weft or warp direction in case of weaved fabrics), but in some situations the textile might be too disorientated and its shear stiffness ought to be used. Secondly, our belt placement model is built on an extended law of Laplace that depends on the belt tension. The belt has a well-defined geometry, but its tension is given by the spring model proposed earlier and unidirectional stretch lines. So far, some local effects, related to structure changes such as the presence of whalebones are not described by this model. Moreover, the body deformation calculated by the Love approach should be homogeneous, semi-infinite and linear elastic. The body stiffness has not been studied in detail here, because a previous study showed that this parameter had little influence on trunk mechanics [22, 34], but a more detailed approach is necessary when considering bones close to the skin surface (iliac crest, ribs). Last, the adhesion is taken into account here by setting the tangential forces to their maximum value before sliding; therefore, our results correspond to a 'worst case' scenario and the real situation lies between totally adhesive and totally sliding behaviour. This model is therefore a first attempt, and more detailed contact behaviour is expected in the future. The combination of a better contact description and long-term material behaviour will pave the way for more complex simulations of gestures such as leaning forward, or moving from sitting to standing.

## Conclusion

This paper describes a complete mechanical model of lumbar belt used in the low-back pain treatment. The model incorporates for the first time deformation of the supports and friction to the classical Law of Laplace to describe the belt effect on trunks of chronic low back pain patients. A unique indicator of the belt mechanical efficiency is proposed: pressure is integrated into a bending moment characterizing the reduction of the lumbar lordosis angle provided by the belt.

A first in-silico trial of two characteristic belts is successfully performed and two original results are outlined. Firstly, the model shows the great influence of the patient’s morphology (characterized by the BMI) on the belt action and manufactured lumbar belts for high BMI patients ought to be re-designed. Secondly, the sticking effect clearly modifies the pressure distribution, with a possible influence on the patient comfort. From this point of view, the belt kinematics during closure is an important design parameter that perturbs dramatically the pressure distribution.

## Supporting information

S1 File Supporting information. The ZIP file contains rough data corresponding to the tensile tests and body shape. All data are provided in CSV file format.
(ZIP)
